# Youth Firearm Mortality in the Americas From 2015 to 2022

**DOI:** 10.1001/jamanetworkopen.2024.37395

**Published:** 2024-10-02

**Authors:** Michelle Degli Esposti, Christian D. Pulcini, Eric William Fleegler, Eugenio Weigend Vargas, Laura Vargas, Adriana Yock-Corrales, Stephen Hargarten

**Affiliations:** 1 Institute for Firearm Injury Prevention, University of Michigan, Ann Arbor; 2 Department of Emergency Medicine and Pediatrics, University of Vermont Medical Center and Children’s Hospital, University of Vermont Larner College of Medicine, Burlington; 3 Departments of Pediatrics, Harvard Medical School, Boston, Massachusetts; 4 Department of Emergency Medicine, Harvard Medical School, Boston, Massachusetts; 5 Department of Emergency Medicine, Massachusetts General Hospital, Boston; 6 Department of Psychiatry, University of Colorado School of Medicine, Denver; 7 Department of Emergency Medicine, Hospital Nacional de Niños “Dr Carlos Saenz Herrera,” CCSS, San José, Costa Rica; 8 Department of Emergency Medicine, Medical College of Wisconsin, Milwaukee

## Abstract

This cross-sectional study uses national vital statistics on firearm mortality to investigate trends in firearm deaths among youths in the US, Brazil, Mexico, and Colombia.

## Introduction

Global firearm injuries cause approximately 28 000 youth deaths per year, with over 60% of injuries affecting youths in the Americas.^1^ Recently, firearm injury was identified as the leading cause of death among US youths,^2^ paralleling data from Mexico, Brazil, and Colombia.^3,4,5^ Comprehensive, up-to-date, comparable trends on firearm mortality in these countries is less well documented. Given the unknown collective burden of firearm deaths among youths across the Americas, we describe trends in these 4 countries.

## Methods

We extracted annual national vital statistics on firearm mortality among youths aged 1 to 19 years from 2015 through 2022 in the US (National Center for Health Statistics), Mexico (Instituto Nacional de Estadística y Geografía), Brazil (Sistema de Informação sobre Mortalidade), and Colombia (Departamento Administrativo Nacional de Estadística)—countries with highest numbers of firearm deaths and available mortality data (eMethods in Supplement 1).^1,3^ We identified firearm mortality using *International Classification of Diseases, Tenth Revision, Clinical Modification* codes for firearm homicide (X93-X95) and suicide (X72-X74) and unintentional (W32-W34) and undetermined (Y22-Y24) deaths. We derived age-specific rates per 100 000 person-years, mapped trends, and used descriptive statistics to summarize changes in youth firearm deaths nationally and sub-nationally (states, administrative departments). The University of Michigan Institutional Review Board approved this cross-sectional study and waived informed consent waived, as the study was not human participant research. We followed the STROBE reporting guideline and analyzed data using R, version 4.3.2 (R Project for Statistical Computing).

## Results

**Figure 1.**
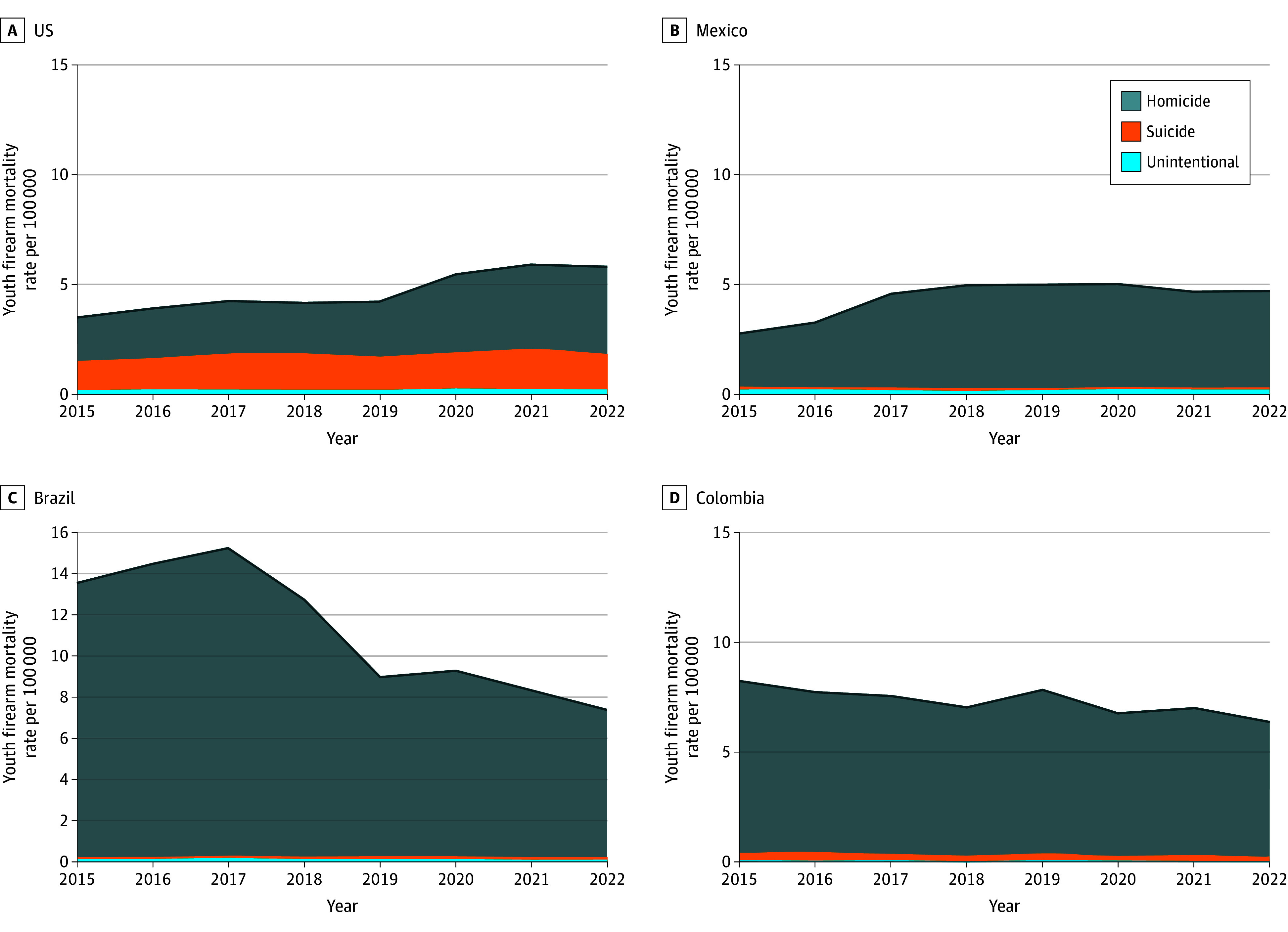
Trends in Youth Firearm Mortality Rates in the US, Mexico, Colombia, and Brazil, 2015 to 2022 Youth is defined as a person aged 1 to 19 years. Firearm deaths are disaggregated by intent: homicide, suicide, and unintentional.

**Figure 2.**
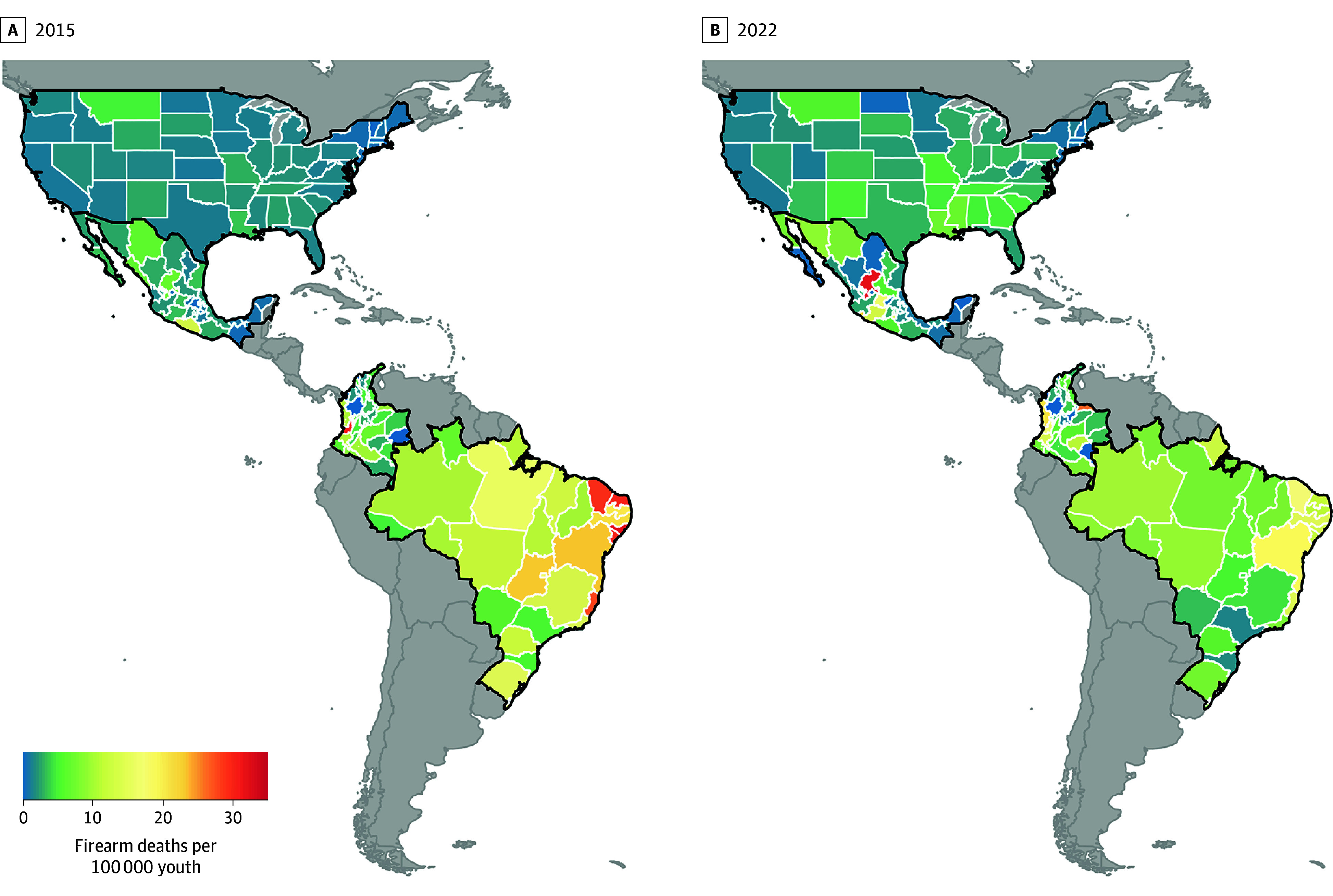
Geographic Variation in Youth Firearm Mortality Rates in 2015 and 2022 Across the Americas Youth is defined as a person aged 1 to 19 years. The gray-shaded region represents no data, as these countries were not included in this study.

From 2015 to 2022, 111 993 youths (101 250 males [90.41%]) died from firearm injuries across the US (29 608), Mexico (15 392), Colombia (9019), and Brazil (57 974). Overall youth firearm mortality rates decreased from 7.07 per 100 000 youths in 2015 to 6.27 per 100 000 youths in 2022. Reductions were not distributed equally; youth firearm mortality rates increased in the US and Mexico and decreased in Colombia and Brazil. In 2015 and 2022, firearm mortality rates per 100 000 person-years were 3.57 vs 5.92 in the US, 2.90 vs 4.88 in Mexico, 8.44 vs 6.52 in Columbia, and 13.81 vs 7.64 in Brazil (Figure 1). There was substantial heterogeneity in the number of firearm deaths per 100 000 youths from 2015 to 2022 (Figure 2). For example, Brazil accounted for 62.60% (8917 of 14 240) of all youth firearm deaths in the region in 2015 but only only 37.40% (4550 of 12162) in 2022.

## Discussion

Despite encouraging reductions in youth firearm mortality in Brazil and Colombia, these have been offset by increases in Mexico and the US. Consequently, the Americas continue to have the highest number of firearm deaths vs any other region, highlighting the need for a public health and policy approach to address the collective burden of youth firearm deaths in the Americas.

Although it is unclear whether specific policies in Brazil and Colombia are associated with reductions in youth firearm deaths, national policies reducing firearm availability (eg, dismantling the Revolutionary Armed Forces of Colombia, Disarmament Statute in Brazil) and citywide restrictions on firearm carriage (Bogotá and Medellín, Colombia; São Paulo, Brazil) may contribute to downward trends. It is also unclear why youth firearm deaths in the US and Mexico continue to increase, although increased firearm availability may be a factor. Firearms are a leading cause of preventable premature death across the Americas and are regionally intertwined through the flow of legal and illegal firearms. As evidence shows that US policies affect firearm violence beyond its borders, especially in Mexico,^6^ more research is needed to understand how youths are affected by domestic and foreign policies and whether transnational collaboration and coordinated policy responses may prevent youth firearm deaths in the region.

Study limitations include the exclusion of other high burden countries (eg, Venezuela) due to a lack of reliable mortality data during the study period. Cross-national differences in death registration coverage and coding practices should also be considered when interpreting these data, along with the descriptive epidemiological study design that precludes causal inference.

## Notes

Supplement 1.
eMethods.
**eTable.** Number of Total Firearm Deaths (all ages) in the Americas in 2021 by Country
eReferences

Supplement 2.
Data Sharing Statement
