# Supplementary material for: Youth Firearm Mortality in the Americas From 2015 to 2022
Source: JAMA Netw Open. 2024 Oct 2;7(10):e2437395. doi: 10.1001/jamanetworkopen.2024.37395 (PMC11447584; doi:10.1001/jamanetworkopen.2024.37395)
Supplement: Supplement 1. — eMethods. eTable. Number of Total Firearm Deaths (all ages) in the Americas in 2021 by Country eReferences [file jamanetwopen-e2437395-s001.pdf]

Supplementary Online Content

Degli Esposti MD, Pulcini CD, Fleegler EW, et al. Youth firearm mortality in the Americas. *JAMA Netw Open.* 2024;7(10): e2437395.
doi:10.1001/jamanetworkopen.2024.37395

eMethods.

eTable. Number of Total Firearm Deaths (all ages) in the Americas in 2021 by Country

eReferences

This supplementary material has been provided by the authors to give readers additional information about their work.

© 2024 Degli Esposti MD et al. *JAMA Network Open.*

eMethods

Context and Countries

We aimed to map trends in youth firearm mortality in the highest burden countries in the Americas. We defined burden as contributing the highest (absolute) numbers of firearm deaths (homicide, suicide, and unintentional deaths) among all ages, according to the most recent release of the Global Burden of Disease Study data in 2021.[1]

In 2021, there was an estimated total of 242,331 firearm deaths globally. Of which, the region of the Americas contributed over 60% of all firearm deaths. The number of firearm deaths varied widely by country in this world region, with over 55% of all firearm deaths occurring in the top five countries in the Americas (see eTable 1). We therefore aimed to include the top five highest burden countries in the Americas, where data allowed.

Brazil, USA, Mexico, and Colombia had high quality mortality data which was available for the gull study period, 2015 through to 2022 (see Data Sources and Measures below). However, national mortality data for Venezuela during this period were not available due to the release of official government data being paused for several years.[2] Our resulting study sample thus included the top four countries in the Americas that had available complete mortality data during the study period (eTable 1), which is also consistent with prior studies documenting trends in firearm mortality across the Americas.[3]

Data Sources and Quality

We used the following data sources to extract youth firearm mortality data for the USA (National Center for Health Statistics),[4] Mexico (Instituto Nacional de Estadística y Geografía),[5] Brazil (Sistema de Informação sobre Mortalidade),[6] and Colombia (Departamento Administrativo Nacional de Estadística).[7] Each country uses a similar process of identifying and defining the underlying cause of death. Specifically, the underlying cause of death is coded according to the tenth revision of the International Classification of Diseases (ICD-10) during routine medical death certification.

The completeness of mortality data for all four countries since 2015 is high.[9] Over 99% of all deaths in the USA and Mexico are registered and medically certified, and over 98% of all deaths in Brazil and Colombia.[3,8] In addition, the GBD have assessed the data quality (using a 5 star based system), and all four countries scored at least 4 stars or above, with more than 80% of all deaths being well certified.[9]

© 2024 Degli Esposti MD et al. *JAMA Network Open.*

eTable. Number of total firearm deaths (all ages) in the Americas in 2021 by Country

Rank	Country in the Americas	Total firearm deaths	Included in study target sample	Excluded due to data availability
1	Brazil	41,046	Yes	No
2	United States of America	40,222	Yes	No
3	Mexico	30,701	Yes	No
4	Venezuela (Bolivarian Republic of)	12,448	Yes	Yes
5	Colombia	10,452	Yes	No
6	Guatemala	3,556	No	…
7	El Salvador	2,669	No	…
8	Argentina	2,318	No	…
9	Honduras	2,125	No	…
10	Canada	926	No	…
11	Peru	893	No	…
12	Ecuador	822	No	…
13	Paraguay	607	No	…
14	Costa Rica	475	No	…
15	Chile	437	No	…
16	Panama	433	No	…
17	Bolivia (Plurinational State of)	413	No	…
18	Uruguay	372	No	…
19	Nicaragua	297	No	…
20	Greenland	10	No	…

Data based on the most recent release of the Global Burden of Disease Study data in 2021.[1]
Total firearm deaths for all ages and both sexes (male and female) in 2021 for firearm homicide, firearm suicide, and unintentional deaths by firearm.

© 2024 Degli Esposti MD et al. *JAMA Network Open.*

eReferences

1. Global Burden of Disease Collaborative Network. Global Burden of Disease Study 2021 (GBD 2021) Results. Published online 2022. Accessed August 1, 2024. https://vizhub.healthdata.org/gbd-results/

2. Lancet T. The collapse of the Venezuelan health system. *Lancet*. 2018;391(10128):1331. doi:10.1016/S0140-6736(16)00277-4

3. Dare AJ, Irving H, Guerrero-López CM, et al. Geospatial, racial, and educational variation in firearm mortality in the USA, Mexico, Brazil, and Colombia, 1990–2015: a comparative analysis of vital statistics data. *The Lancet Public Health*. 2019;0(0). doi:10.1016/S2468-2667(19)30018-0

4. National Center for Health Statistics. National Vital Statistics System: Restricted-Use Multiple Cause of Death Data File, 1989-2019. https://www.cdc.gov/nchs/nvss/dvs_data_release.htm

5. de Salubridad General C. Instituto Nacional de Estadística y Geografía Avisos Indice en página 125. Published online 2016.

6. DATASUS - SIM - Sistema de Informação sobre Mortalidade. Accessed March 20, 2023. http://sim.saude.gov.br/default.asp

7. De Hogares GEI. Departamento Administrativo Nacional de Estadística. Published online 2007.

8. Costa LFL, de Mesquita Silva Montenegro M, Rabello Neto D de L, et al. Estimating completeness of national and subnational death reporting in Brazil: application of record linkage methods. *Population Health Metrics*. 2020;18(1):22. doi:10.1186/s12963-020-00223-2

9. Naghavi M, Marczak LB, Kutz M, et al. Global Mortality From Firearms, 1990-2016. *JAMA*. 2018;320(8):792-814. doi:10.1001/jama.2018.10060

© 2024 Degli Esposti MD et al. *JAMA Network Open.*
