# Supplementary material for: Youth Firearm Mortality in the Americas From 2015 to 2022
Source: JAMA Netw Open. 2024 Oct 2;7(10):e2437395. doi: 10.1001/jamanetworkopen.2024.37395 (PMC11447584; doi:10.1001/jamanetworkopen.2024.37395)
Supplement: Supplement 2. — Data Sharing Statement [file jamanetwopen-e2437395-s002.pdf]

Data Sharing Statement

Degli Esposti. Youth Firearm Mortality in the Americas From 2015 to 2022. *JAMA Netw Open*. Published October 02, 2024. doi:10.1001/jamanetworkopen.2024.37395

Data
Data available: No

Additional Information
Explanation for why data not available: The data are already publicly available from the primary data sources. Upon request to corresponding author, we will provide the aggregated data used in this research letter.
